# Transcriptomic and proteomic analyses of *Cucurbita ficifolia* Bouché (Cucurbitaceae) response to *Fusarium oxysporum* f.sp. *cucumerium*

**DOI:** 10.1186/s12864-022-08674-7

**Published:** 2022-06-13

**Authors:** Junjun Xie, Yumei Ding, Ting Gao, Shuilian He, Kai Zhao, Xuehu Yang, Jie Zhang, Zhengan Yang

**Affiliations:** 1 College of Horticulture and Landscape, Yunnan Agricultural University, Kunming, 650201 Yunnan China; 2 College of Food Science and Technology, Yunnan Agricultural University, Kunming, 650201 Yunnan China; 3 Biotechnology and Germplasm Resources Institute Yunnan Academy of Agricultural Sciences, Kunming, 650205 Yunnan People’s Republic of China

**Keywords:** *Cucurbita ficifolia* Bouché, FOC, iTRAQ, RNA-sequencing, Defense response

## Abstract

**Background:**

*Fusarium oxysporum* f. sp. *cucumerinum* (FOC) is the causal agent of cucumber Fusarium wilt, which can cause extensive damages and productivity losses. *Cucurbita ficifolia* Bouché (Cucurbitaceae) is usually used as rootstock for cucumber because of its excellent resistance to Fusarium wilt. Our previous study found that *C*.*ficifolia* has high FOC resistance, the underlying mechanism of which is unclear.

**Results:**

Transcriptome and proteome profiling was performed on the basis of RNA-Seq and isobaric tag for relative and absolute quantitation technology to explore the molecular mechanisms of the response of *Cucurbita ficifolia* Bouché to *Fusarium oxysporum* f. sp. *cucumerium* infection. Comparative analyses revealed that 1850 genes and 356 protein species were differentially regulated at 2d and 4d after FOC inoculation. However, correlation analysis revealed that only 11 and 39 genes were differentially regulated at both the transcriptome and proteome levels after FOC inoculation at 2d and 4d, respectively. After FOC inoculation, plant hormones signal transduction, transcription factors were stimulated, whereas wax biosynthesis and photosynthesis were suppressed. Increased synthesis of oxidative-redox proteins is involved in resistance to FOC.

**Conclusions:**

This study is the first to reveal the response of *C*. *ficifolia* leaf to FOC infection at the transcriptome and proteome levels, and to show that FOC infection activates plant hormone signaling and transcription factors while suppressing wax biosynthesis and photosynthesis. The accumulation of oxidative-redox proteins also plays an important role in the resistance of *C. ficifolia* to FOC. Results provide new information regarding the processes of *C*. *ficifolia* leaf resistance to FOC and will contribute to the breeding of cucumber rootstock with FOC resistance.

**Supplementary Information:**

The online version contains supplementary material available at 10.1186/s12864-022-08674-7.

## Background

*Fusarium oxysporum* f. sp. *cucumerinum* (FOC) is the causal agent of cucumber Fusarium wilt which can cause serious economic losses, limit production, and decrease fruit quality. FOC infects cucumber through the roots and rapidly invades the aboveground parts via vascular tissues, thus resulting in plant water and nutrient transport blockage and plant wilt [1, 2]. Fusarium wilt is difficult to control, because it is aggravated by intensive farming practices and FOC can survive in the soil for several years [3, 4]. Effective management approaches for controlling cucumber wilt include the use of resistant cultivars, germicides, and grafting to resistant rootstock. However, FOC-resistant varieties are difficult to cultivate, grafting to resistant rootstock is widely used for cucumber to limit the effects of soil-borne pathogens in winter greenhouses and under protected structures [5, 6].

*Cucurbita ficifolia* Bouché (Cucurbitaceae) is a species of *Cucurbita*. It originated from Central America and South America, and is thus far mainly cultivated in the low latitude plateau areas, such as Yunnan in China. The color of its mature seeds is black. Great variability is observed in the seed colors of other *Cucurbita* speies. *C. ficifolia* which is also called as ‘Black Seeded’ figleaf gourd is usually used as the rootstock for cucumber because of its excellent resistance to Fusarium wilt and salt stress [7, 8].

Pathogen infection triggers complex signaling networks in plant cells. Salicylic acid (SA), jasmonic acid (JA)and ethylene (ET) are the main phytohormones related to host–pathogen interactions; and they modulate each other through a complex network of regulatory interactions [9–11]. Abscisic acid (ABA) and auxin are also key components of the immune response of plants [12]. The ABA content in wilted cucumber plants is higher than that healthy ones after infection with FOC, and ABA may play a crucial regulatory role in modifying stomatal behavior, that results in cucumber wilting due to water loss [13, 14]. *F. oxysporum* f. sp. *melonis* race 1.2(FOM1.2) is the most virulent and yield-limiting pathogen of melon. The melon genotype NAD is highly resistant to FOM1.2. Transcriptome analysis revealed that the resistance of genotype NAD is mainly signaled by JA and ET pathways mediated by ABA and auxin after FOM1.2 infection [15]. After phytohormones signaling is activated, downstream transcription factors (TFs) are also triggered, causing changes in the expression of related genes [16, 17]. TFs orchestrate the dynamic interplay between defense genes and the biosynthesis of chemical metabolites during host–pathogen interaction [16].

Next-generation sequencing technology is widely used to systematically reveal plant responses to biotic stresses. It has enriched the knowledge on mRNAs in multiple adverse environments [18]. Proteome profiling can reveal the dynamics of proteins, post-translational modifications, and biological pathways in plants in response to biological stress [19]. The complementary transcriptome and proteome analysis is widely used to resolve plant responses to various biotic stresses. *Cylindrocladium* leaf blight caused by *Calonectria pseudoreteaudii* is one of the most severe diseases in *eucalyptus* plantations and nurseries. The combined transcriptome and proteome analysis of the leaves of resistant *eucalyptus* cultivars revealed that the JA and sugar signaling pathways were activated after *C. pseudoreteaudii* infection, whereas photosynthesis and protein metabolism were suppressed [20]. *Ciboria carunculoides* is one of the disease pathogens that are most relevant to the economic and field losses of mulberry fruit in China. The combined transcriptome and proteome analyses of mulberry fruit at the early and middle stages of *C. carunculoides* infection revealed that plant hormone signaling pathways, TFs, and secondary metabolites were stimulated, whereas photosynthesis and cellular growth-related metabolism were inhibited [21]. However, studies on the response to *C. ficifolia* to *F. oxysporum* have not been reported.

The transcriptome and proteome of *C. ficifolia* infected with FOC were comparatively analysed by using RNA-seq and isobaric tag for relative and absolute quantitation technology. Comprehensive genome-wide analyses uncovered several interesting insights into *C. ficifolia* and FOC interactions. This work could further provide a reference for the rootstock breeding of cucumber with fungal pathogen resistance.

## Results

### Overview of transcriptome and proteome analyses

Given the unavailability of the genomic data of *C. ficifolia* and the limited read length of the Illumina-seq platform, we first used PacBio sequencing to splice the reference genome. A total of 62 169 unigenes with an average length of 1160 nt were obtained. Sequence alignment by using BLAST showed that 40 031 (64.39%) transcripts were exhibited gene annotation. The unigenes were aligned to the COG database to predict their possible functions. Gene ontology (GO) functional annotations were obtained accordance with Nr annotation information.

**Fig. 1:**
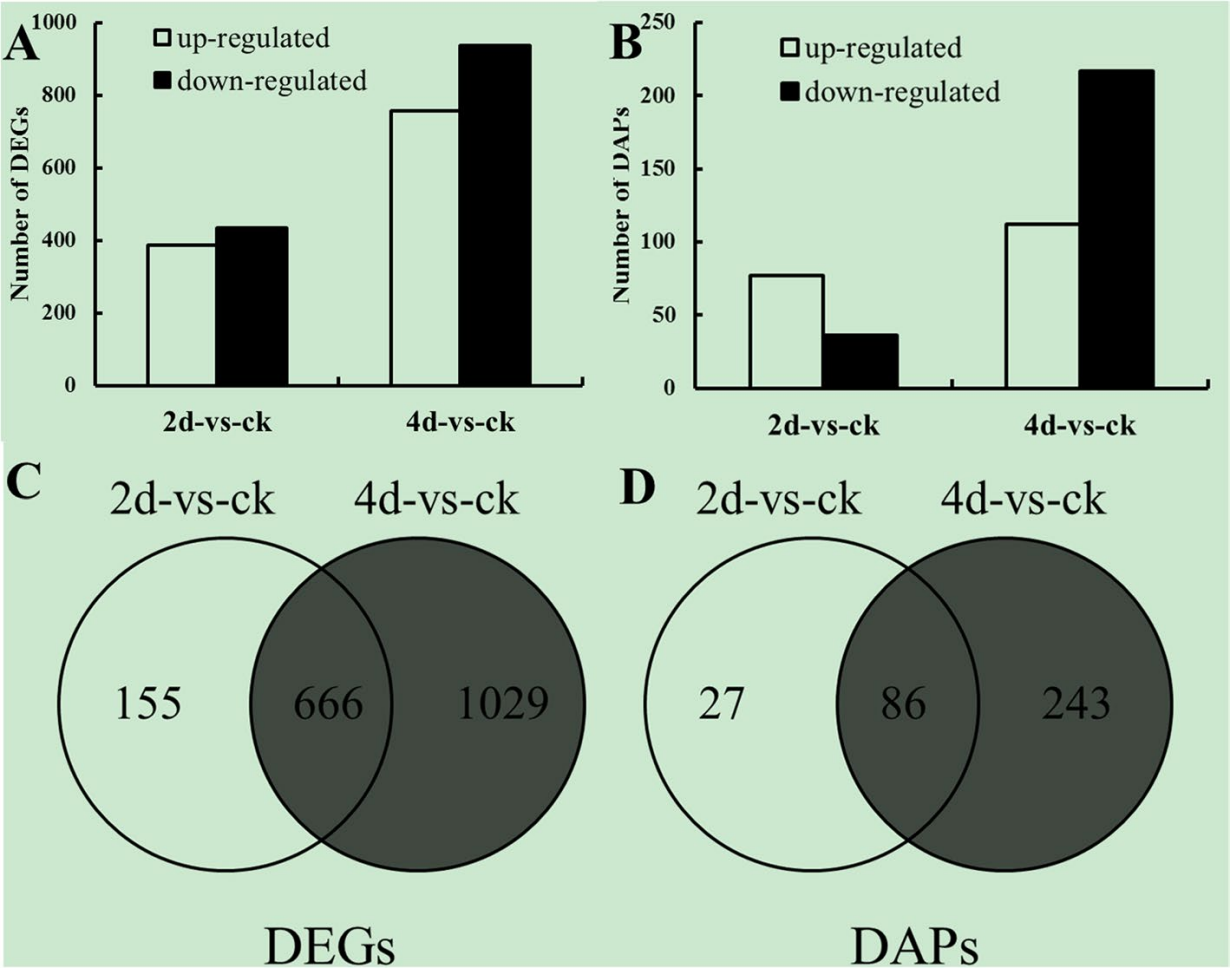
DEGs and DAPsin *C. ficifolia* leaf after infection by FOC. Number of DEGs (**A**) and DAPs (**B**) at 2d and 4d compared with control. Venn diagrams showing DEGs (**C**) and DAPs (**D**) classified by their expression pattern

Approximately 48 million clean reads were obtained from each Illumina-seq sample. Genes with expression changes of no less than 2-folds (log2 ratio ≥ 1) and false discovery rate (FDR) < 0.05 were identified as differentially expressed genes (DEGs). In total, 1850 genes in C. *ficifolia* leaves were found to be differentially expressed after FOC infection. A total of 821 (387 up-regulated and 434 down-regulated) and 1695 (758 up-regulated and 937down-regulated) DEGs were identified to be responsive to FOC infection in 2d-vs-ck and 4d-vs-ck, respectively (Fig. 1; Additional files 1 and 2). Of the 1850 DEGs, 666 were affected by FOC infection in both 2d-vs-ck and 4d-vs-ck with consistent trends (both up- regulated or down—regulated), (Fig. 1).

Comparative proteomic analyses were performed on *C. ficifolia* leaf samples at 2 and 4 d post infection (dpi) and the control treatment. A total of 231 157 spectra were obtained, in which 62 490 distinct peptides, and 2491 protein species were identified. Protein species with a log2 ratio ≥ 1 and FDR < 0.05 were identified as differentially accumulated proteins (DAPs). In *C. ficifolia* leaves, 356 protein species showed differential expression after FOC infection. A total of 113 (77 up-regulated and 36 down-regulated) and 329 (112 up-regulated and 217 down-regulated) DAPs were identified to be responsive to FOC infection in 2d-vs-ck and 4d-vs-ck respectively (Fig. 1; Additional files 3 and 4). Of the 356 DAPs, only 86 were regulated by FOC in both 2d-vs-ck and 4d-vs-ck (Fig. 1; Additional files 3 and 4).

### Phytohormone signal transduction pathways and TFs involved in defense of C. ficifolia at the transcriptional level

Of the 666 DEGs affected by FOC infection in both 2d-vs-ck and 4d-vs-ck, more than twenty genes were related to plant hormone signaling pathways. Nine genes associated with ET signaling were differentially regulated by FOC infection. Among these genes, four were ethylene-responsive TFs, of which three were up-regulated; one, namely 1-aminocyclopropane-1-carboxylate oxidase, was up-regulated. Seven genes involved in auxin signaling, except a gene encoding auxin-responsive protein, were both up-regulated by FOC infection in 2d-vs-ck and 4d-vs-ck. The ABA receptor PYL4 was up-regulated at the mRNA level in 2d-vs-ck and 4d-vs-ck, and Protein MOTHER of FT and TFL1 which respond to ABA were also up-regulated in 2d-vs-ck and 4d-vs-ck. Two genes respond to JA or cytokinin, respectively, also showed consistent trend in 2d-vs-ck and 4d-vs-ck (Table 1). Twelve DEGs (six up- regulated and six down-regulated) in both 2d-vs-ck and 4d-vs-ck were annotated as transcription factors, with most belong to MYB, bHLH, LUX and GLK TF families (Table 1). Four resistance-related genes, including universal stress protein A-like protein, isochorismate synthase, protein ENHANCED DISEASE RESISTANCE 4 and wound-induced protein were both up-regulated in 2d-vs-ck and 4d-vs-ck (Table 1).

**Table 1:** DEGs in C. *ficifolia* leaf after infection by FOC at 2 dpi and 4 dpi

Gene ID	2d-VS-CK log2FC	4d-VS-CK log2FC	Gene annotation
**Ethylene**			
CL780Contig2	-4.07	-1.78	Protein REVEILLE 7, response to ethylene
comp69483_c1_seq15_1	-3.57	-3.06	Protein REVEILLE 7, response to ethylene
CL4715Contig1	-1.89	-2.01	Protein REVEILLE 6, response to ethylene
comp43670_c0_seq2_3	6.05	6.41	Protein REVEILLE 6, response to ethylene
CL28656Contig1	2.42	2.76	Ethylene-responsive transcription factor ERF106
CL42540Contig1	2.72	2.40	Ethylene-responsive transcription factor ERF106
CL27086Contig1	3.08	3.28	Ethylene-responsive transcription factor ERF053
CL21678Contig1	-2.90	-2.50	Ethylene-responsive transcription factor 4
CL3070Contig1	2.13	2.95	1-aminocyclopropane-1-carboxylate oxidase homolog 3
**Auxin**			
CL30348Contig1	-2.21	-3.69	Auxin-responsive protein SAUR50
CL1073Contig1	2.17	3.11	Auxin-repressed 12.5 kDa protein
CL2472Contig1	2.28	3.19	Auxin-repressed 12.5 kDa protein
CL24742Contig1	1.92	1.82	Auxin response factor 6
comp51974_c0_seq1_1	2.08	2.02	Auxin response factor 6
CL7285Contig1	2.02	2.64	IAA-amino acid hydrolase ILR1,auxin metabolic process
CL24970Contig1	3.42	4.92	Glutathione S-transferase,auxin-activated signaling pathway
**Abscisic acid**			
CL10966Contig1	3.91	3.15	Protein MOTHER of FT and TFL1,response to abscisic acid
CL24926Contig1	2.06	1.76	Abscisic acid receptor PYL4
**Other hormones**			
comp63305_c0_seq6_2	2.51	2.35	Protein NRT1/PTR FAMILY 6.2,response to jasmonic acid
CL621Contig1	-2.39	-3.04	Cytokinin dehydrogenase 1
**Transcription factor**			
CL4801Contig1	2.46	2.57	Transcription factor SRM1
comp63308_c1_seq4_1	1.88	2.22	Transcription factor SRM1
CL2579Contig1	7.78	7.76	Transcription factor PCL1
CL24093Contig1	-2.25	-3.34	Transcription factor MYB1R1
CL26072Contig1	4.77	5.46	Transcription factor LUX
CL37562Contig1	6.00	6.98	Transcription factor LUX
comp62782_c2_seq8_2	-1.83	-1.57	Transcription factor ILR3
CL1Contig37	-5.03	-5.08	Transcription factor HY5
comp75510_c0_seq1_2	2.14	2.00	Transcription factor BOA
CL51358Contig1	-2.70	-2.82	Transcription factor bHLH66
CL19223Contig1	-2.42	-1.96	Transcription activator GLK1
CL1942Contig2	-2.21	-1.89	Transcription activator GLK1
**Resistance-related genes**			
CL25265Contig1	3.62	4.36	Universal stress protein A-like protein
CL478Contig1	3.32	4.82	Isochorismate synthase, chloroplastic
comp67592_c0_seq158_1	2.47	1.95	Isochorismate synthase, chloroplastic
CL29528Contig1	3.87	4.57	Protein ENHANCED DISEASE RESISTANCE 4
CL601Contig2	1.95	2.15	Wound-induced protein 1
**Photosynthesis**			
CL5872Contig1	-2.77	-2.97	Photosystem II 22 kDa protein, chloroplastic
CL8698Contig1	-2.29	-2.76	Photosystem II 22 kDa protein, chloroplastic
CL50394Contig1	-1.81	-1.77	Magnesium-protoporphyrin IX monomethyl ester cyclase
CL4989Contig1	-2.15	-3.37	Magnesium-chelatase subunit ChlH, chloroplastic
CL25245Contig1	-1.73	-2.31	Chlorophyll a-b binding protein 13, chloroplastic

A total of 1029 DEGs were found in 4d-vs-ck. However, 666 DEGs were affected by FOC infection in both 2d-vs-ck and 4d-vs-ck and were enriched in plant hormone signal transduction pathways, TFs and resistance-related. Most of the transcript species related to the plant hormone signal transduction pathway were involved in ET, auxin, ABA, and JA signaling. A gene responsive to brassinosteroids was also down-regulated in 4d-vs-ck (Table 2). Fifteen DEGs (nine up-regulated and six down-regulated) were annotated as TFs in 4d-vs-ck (Table 2), with most belonging to the MYB, WARK, TCP and NAC TF families. Seventeen resistance-related genes were found in 4d-vs-ck. They included disease resistance, pathogenesis-related and TMV resistance proteins. Two DEGs annotated as pathogenesis-related proteins showed increased up-regulation with the log2FC of 8.3 and 9.0.

**Table 2:** DEGs in C. *ficifolia* leaf after infection by FOC only at 4 dpi

Gene ID	4d-VS-CK log2FC	Gene annotation
**Ethylene**		
CL12896Contig1	3.92	Ethylene-responsive proteinase inhibitor 1
comp51941_c0_seq1_3	-1.50	Ethylene receptor 2
CL23139Contig1	1.94	1-aminocyclopropane-1-carboxylate oxidase 5
**Jasmonic acid**		
CL19963Contig1	2.59	3-ketoacyl-CoA thiolase 2, peroxisomal
CL21247Contig1	2.56	3-ketoacyl-CoA thiolase 2, peroxisomal
**ABA**		
comp51694_c0_seq3_2	2.01	ABSCISIC ACID-INSENSITIVE 5-like protein 6
CL23454Contig1	2.10	Abscisic acid receptor PYL8
CL23796Contig1	-1.81	Abscisic acid receptor PYL4
**Auxin**		
CL46582Contig1	-1.51	Auxin-induced protein 22D
CL38635Contig1	2.05	Auxin transport protein BIG
CL28225Contig1	-4.01	Auxin efflux carrier component 5
**Other hormones**		
CL25910Contig1	1.92	Gibberellin 20 oxidase 1
CL14729Contig1	-1.40	Protein EXORDIUM, response to brassinosteroid
CL30190Contig1	-1.41	Salicylic acid-binding protein 2
**Resistance-related**		
CL5641Contig1	3.37	Adrenodoxin-like protein 2, mitochondrial
CL28332Contig1	-2.41	Peroxidase 47
CL304Contig1	-1.89	Peroxidase 39
CL52011Contig1	3.12	Disease resistance protein RPS6
CL33669Contig1	1.36	MLO-like protein 6
CL4874Contig1	1.25	MLO-like protein 12
CL18414Contig1	-3.36	Pathogenesis-related protein PR-1
CL26231Contig1	8.30	Pathogenesis-related protein P2
CL10878Contig1	9.06	Pathogenesis-related protein 1
CL1932Contig1	3.14	Pathogenesis-related genes transcriptional activator PTI6
CL51123Contig1	-1.27	Protein ENHANCED DISEASE RESISTANCE 2-like
CL8780Contig1	-2.77	Putative disease resistance RPP13-like protein 1
CL512Contig2	-1.12	S-norcoclaurine synthase 2
comp53156_c0_seq3_1	1.57	Wound-induced protein 1
CL48028Contig1	1.94	Universal stress protein A-like protein
CL19588Contig1	1.52	TMV resistance protein N
CL21402Contig1	2.24	TMV resistance protein N
**Transcription factor**		
CL30475Contig1	1.40	MYB family transcription factor EFM
CL50886Contig1	1.85	MYB family transcription factor EFM
CL32159Contig1	-1.18	Transcription factor MYB59
comp75186_c0_seq1_2	-2.36	Transcription factor MYB44
CL54017Contig1	4.45	NAC domain-containing protein 79
CL26897Contig1	3.15	Probable WRKY transcription factor 70
CL2070Contig1	3.32	Probable WRKY transcription factor 69
comp64969_c0_seq1_1	1.17	Transcription factor TCP4
CL45926Contig1	2.78	Transcription factor TCP20
CL28172Contig1	-1.71	Transcription factor SRM1
CL25039Contig1	1.86	Transcription factor GTE12
CL38022Contig1	-1.73	Transcription factor EMB1444
comp44643_c0_seq12_3	-1.34	Transcription factor DIVARICATA
CL10732Contig1	-4.66	Transcription factor bHLH92
**Wax biosynthetic process**		
CL13276Contig1	-2.12	3-ketoacyl-CoA synthase 6
CL6623Contig1	-1.52	3-ketoacyl-CoA synthase 6
CL26021Contig1	-1.97	3-ketoacyl-CoA synthase 4
comp31671_c0_seq2_2	-1.77	3-ketoacyl-CoA synthase 2
comp37543_c1_seq2_3	-1.94	3-ketoacyl-CoA synthase 12
CL141Contig1	-1.38	3-ketoacyl-CoA synthase 11
CL51488Contig1	-1.53	3-ketoacyl-CoA synthase 10
CL26445Contig1	-1.62	Acyl-[acyl-carrier-protein] desaturase, chloroplastic
CL23269Contig1	1.87	Long chain acyl-CoA synthetase 4
CL26279Contig1	-1.47	Omega-hydroxypalmitate O-feruloyl transferase
CL778Contig1	-4.27	Omega-hydroxypalmitate O-feruloyl transferase
CL26276Contig1	-2.45	Protein ECERIFERUM 1
CL32066Contig1	-2.54	Protein ECERIFERUM 1
CL1392Contig2	-1.83	Protein HOTHEAD
comp50398_c2_seq5_3	-3.10	Protein HOTHEAD
CL50895Contig1	-1.71	Very-long-chain (3R)-3-hydroxyacyl-CoA dehydratase 2
**Photosynthesis**		
CL42418Contig1	-1.46	Photosystem II reaction center W protein, chloroplastic
CL7255Contig1	-1.06	Photosystem II protein psbY-2, chloroplastic
CL30417Contig1	-1.64	Photosystem II 5 kDa protein, chloroplastic
CL46717Contig1	-12.75	Photosystem I reaction center subunit psaK, chloroplastic
CL21783Contig1	-2.04	Photosystem I reaction center subunit N, chloroplastic
CL22180Contig1	-1.19	Photosystem I reaction center subunit III, chloroplastic
CL782Contig1	-1.60	Photosystem I reaction center subunit III, chloroplastic
comp67722_c4_seq2_2	-1.27	Chlorophyll a-b binding protein of LHCII type I
CL31939Contig1	-1.44	Chlorophyll a-b binding protein of LHCII type 1
CL13308Contig1	-2.15	Chlorophyll a-b binding protein CP29.1, chloroplastic
CL20903Contig1	-2.34	Chlorophyll a-b binding protein CP29.1, chloroplastic
comp69242_c0_seq1_2	-13.19	Chlorophyll a-b binding protein CP29.1, chloroplastic
CL30759Contig1	-2.02	Chlorophyll a-b binding protein CP26, chloroplastic
CL55345Contig1	-1.76	Chlorophyll a-b binding protein CP24 10A, chloroplastic
CL25433Contig1	-1.05	Chlorophyll a-b binding protein 8, chloroplastic
CL11837Contig1	-1.38	Chlorophyll a-b binding protein 7, chloroplastic
CL54964Contig1	-1.28	Chlorophyll a-b binding protein 7, chloroplastic
CL55558Contig1	-13.46	Chlorophyll a-b binding protein 4, chloroplastic
CL23730Contig1	-2.31	Chlorophyll a-b binding protein 3, chloroplastic
CL38334Contig1	-2.17	Chlorophyll a-b binding protein 3, chloroplastic
CL2559Contig1	-1.59	Chlorophyll a-b binding protein 151, chloroplastic
CL2559Contig2	-1.86	Chlorophyll a-b binding protein 151, chloroplastic

### Wax biosynthetic process and photosynthesis are partially repressed by FOC at 4dpi

Among the 1029 DEGs that were up- or down-regulated only in 4d-vs-ck, many related to wax biosynthetic process, carbon fixation and photosynthesis were down-regulated. DEGs annotated as 3-ketoacyl-CoA synthase, long chain acyl-CoA synthase and protein ECERIFERUM, which are related to wax biosynthetic process, were both down-regulated. Several genes encoding key enzymes associated with carbon fixation were down-regulated after FOC infection. These genes included sedoheptulose-1,7-bisphosphatase, glyceraldehyde-3-phosphate dehydrogenase and fructose-1,6-bisphosphatase. A total of 22 down-regulated DEGs were involved in photosynthesis and were related to photosystems I and II and chlorophyll binding proteins. Two DEGs that were annotated as photosystem I reaction center subunit and chlorophyll a-b binding protein were highly down-regulated, with the log2FC of -12.75 and -13.19, respectively (Table 2).

### Oxidative-redox proteins involved in defense of C. ficifolia at the translational level

There were 86 DAPs which regulated by FOC in both 2d-vs-ck and 4d-vs-ck, six proteins were annotated as oxidative-redox proteins (Table 3). Six oxidative-redox proteins were one catalase isozyme, two ferredoxins, and three peroxidases. All of the oxidative-redox proteins showed increased accumulation at 2dpi and 4dpi. Three photosystem proteins, one photosystem I reaction center subunit N and two oxygen-evolving enhancer proteins also were up-regulated after FOC infected. Sixteen ribosomal proteins showed decreased accumulation at 2dpi and 4dpi.

**Table 3:** DAPs in C. *ficifolia* leaf after infection by FOC at 2 dpi and 4 dpi

ID	2d-VS-CK log2FC	4d-VS-CK log2FC	Annotation
Oxidative-redox proteins			
CL21412Contig1	1.59	1.92	Ferredoxin-1, chloroplastic
CL29295Contig1	2.09	2.24	Ferredoxin-2, chloroplastic
CL34362Contig1	2.03	2.98	Catalase isozyme 3
CL22454Contig1	3.47	4.07	Peroxidase 21
CL7963Contig1	1.61	2.01	Peroxidase 15
comp72441_c0_seq1_2	2.06	2.75	Peroxidase 2
Photosystem proteins			
CL21783Contig1	2.05	2.44	Photosystem I reaction center subunit N
CL1Contig19	1.38	1.83	Oxygen-evolving enhancer protein 3–2
comp66617_c2_seq1_2	1.65	2.36	Oxygen-evolving enhancer protein 1
Ribosomal protein			
CL10872Contig1	-1.18	-2.34	60S ribosomal protein L27-3
CL12198Contig1	-1.06	-1.36	60S ribosomal protein L19-2
CL22024Contig1	-1.12	-2.17	60S ribosomal protein L24
CL23985Contig1	-1.28	-1.50	40S ribosomal protein S15a-1
CL25275Contig1	-1.03	-1.80	40S ribosomal protein S10-3
CL26010Contig1	-1.10	-1.91	60S ribosomal protein L9
CL27757Contig1	-1.22	-1.75	30S ribosomal protein S3
CL42728Contig1	-1.14	-2.35	60S acidic ribosomal protein P2
CL43344Contig1	-1.13	-1.93	60S ribosomal protein L35
CL43617Contig1	-1.22	-1.84	40S ribosomal protein S13
CL43782Contig1	-1.15	-2.09	40S ribosomal protein S6-2
CL47329Contig1	-1.03	-1.64	60S ribosomal protein L22-2
CL47423Contig1	-1.31	-2.06	40S ribosomal protein S16
CL52891Contig1	-1.89	-3.29	40S ribosomal protein S2-4
CL53777Contig1	-1.15	-2.39	60S ribosomal protein L7a-2
comp72390_c0_seq1_2	-1.11	-1.75	50S ribosomal protein L35, chloroplastic

### Correlation analysis of transcriptome and proteome data

**Fig. 2:**
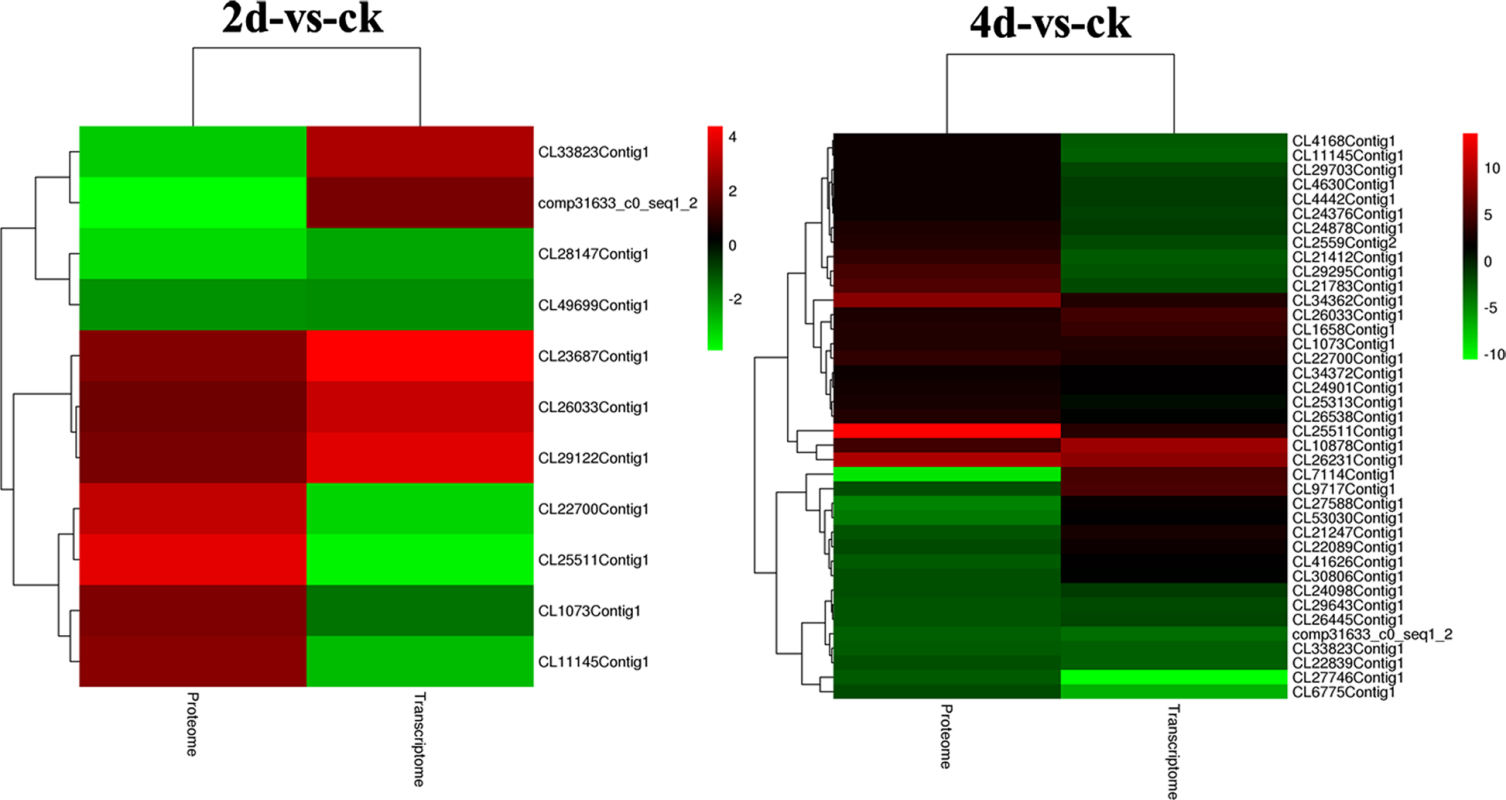
Cluster analysis of the DEGs and DAPs with correlated regulation in the transcriptome and proteome datasets

Transcriptome and proteome data were compared on the basis of the log2-transformed protein species accumulation and log2-gene expression ratios. The DAPs were associated with the corresponding DEGs in accordance with annotations or id correspondences. If they could not be directly related, they were associated in accordance with gene names (protein names) or the BLAST sequences of homologous pairs. A low correlation was observed between transcriptome data and proteome data at 2di and 4di (Fig. 2, Additional files 5 and 6). Compared with control, only 11 genes showed correlated regulation at both the transcription and translation levels at 2dpi. Among these genes, only nine had consistent trends and two had opposite trends at the transcription and translation levels (Additional file 5). At 4 d after FOC infection, 39 genes showed a correlated regulation between transcriptome and proteome data. Of these genes, 19 had the consistent trends and 20 had the opposite trends at the transcription and translation levels (Additional file 6). Two genes that were annotated as pathogenesis-related protein and one gene that was annotated as peroxidase were up-regulated at the transcription and translation levels at 4dpi.

**Fig. 3:**
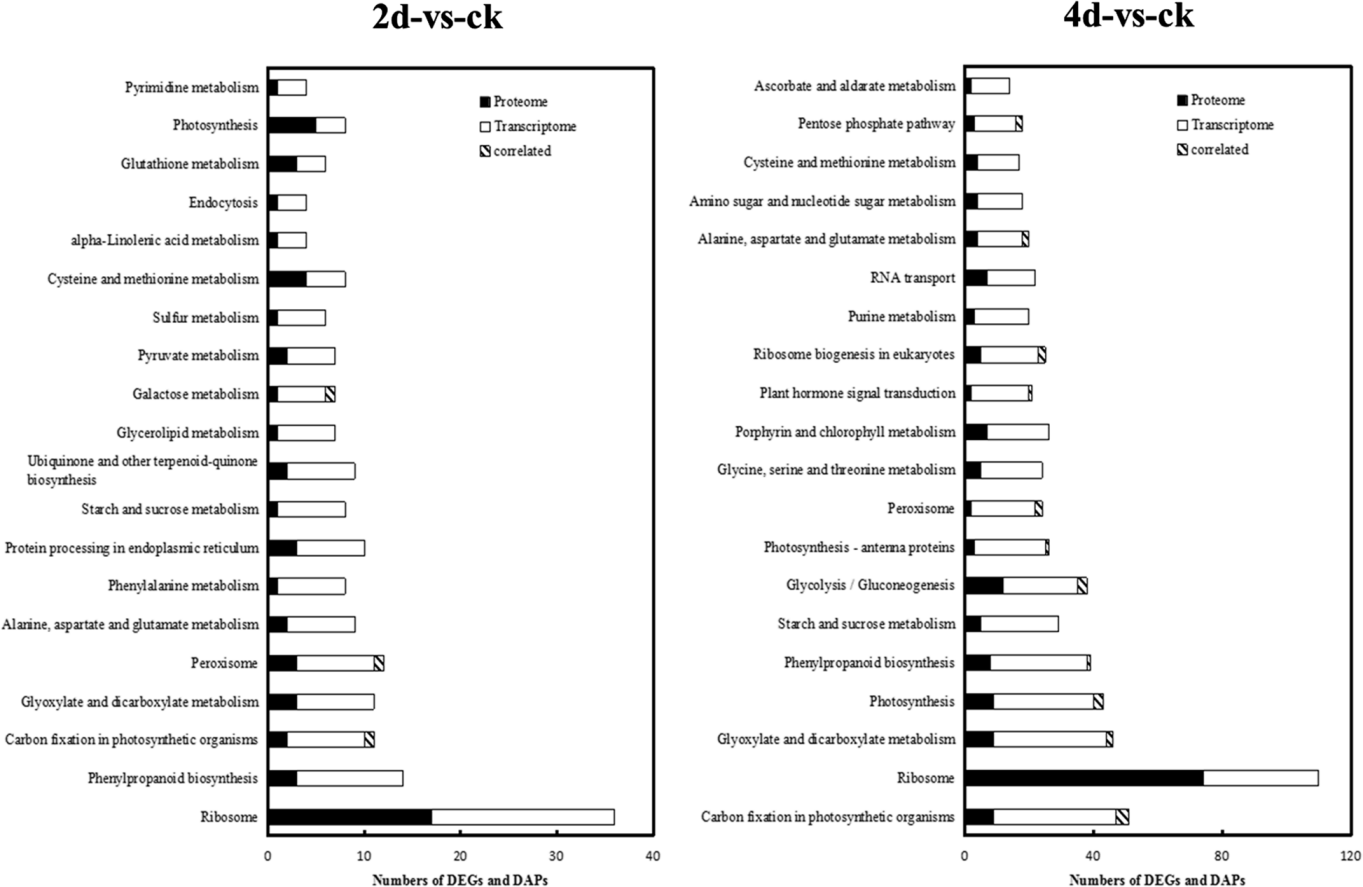
KEGG pathway enrichment analysis of DEGs and DAPs in 2d and 4d infected *C. ficifolia* leaf compared with control

KEGG pathway enrichment analysis revealed that DEGs and DAPs were mainly related to the ribosome, phenylpropanoid biosynthesis, carbon fixation in photosynthetic organisms, peroxisome, glyoxylate and dicarboxylate metabolism, and glycolysis/gluconeogenesis pathways (Fig. 3).

### Real-time polymerase chain reaction validation

**Fig. 4:**
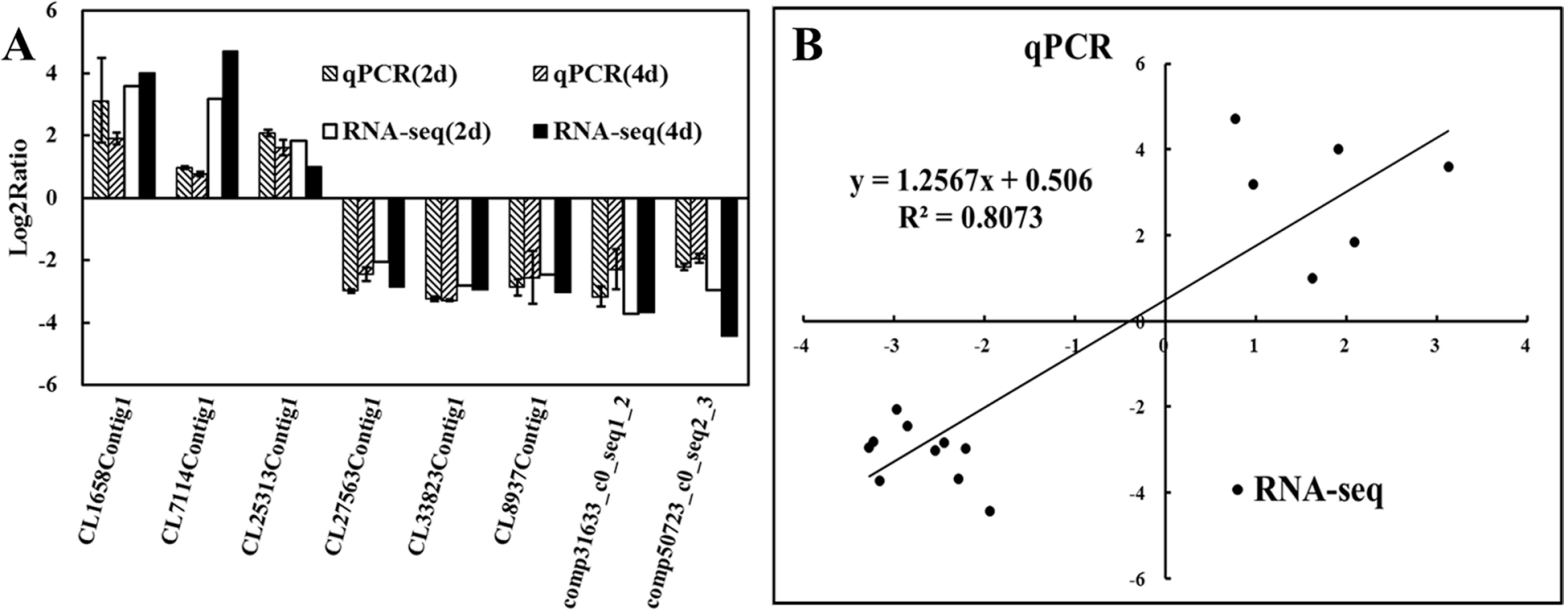
Validation of the transcriptome data. **A** Results from RNA-seq analysis were compared with those from qPCR. Bars represent mean ± SD (*n* = 3). **B** Comparison of log2 expression of 8 selected differentially regulated genes at 2d and 4d infected *C. ficifolia* leaf as measured by RNA-seq and qPCR. Positive and negative log2 expression ratios represent up- and down-regulation, respectively, compared with control

Eight DEGs were selected for real-time polymerase chain reaction analysis to validate the RNA-seq results. These genes showed different expression patterns in *C. ficifolia* leaves at 2 dpi and 4 dpi. The expression patterns of these genes obtained through qRT-PCR confirm to a large extent the transcriptome data (R^2^ = 0.8073; Fig. 4).

## Discussion

Biotic stress, especially pathogen infection, is generally believed to trigger complex molecular changes in plants. The complete image of molecular dynamics can be revealed via transcriptome and proteome profiling [22]. Leaf samples were obtained at different infection stages to investigate the mechanism of *C*. *ficifolia* response to FOC infection.

### Leaf response is critical for resolving FOC resistance in C. ficifolia at early stage

FOC infects plants through the roots and rapidly invades the aboveground parts via vascular tissues, thus resulting in plant water and nutrient transport blockage and wilting. In cucumber, which is sensitive to FOC, leaves exhibited stomata closure, decreased transpiration rate, and water loss that then resulted in cell death, during the early stages of FOC infection [13]. However, at the early stage of FOC infection, *C. ficifolia* showed significantly increased stomatal conductance, transpiration rate and net photosynthetic rate. Our previous study also found that the expression level of HQRGA2, a homologous sequence of NBS disease-resistance gene (Genbank ID: MG946756) significantly increased and declined at 2 and 4dpi [23]. These results indicated that in *C. ficifolia*, the response of the aboveground parts at the early infection stage is the key mechanism of resistance to FOC. We selected leaf samples of *C. ficifolia* with short-term infection (2 and 4 days) for Pacbio and Illumina sequencing to obtain sufficient information regarding the response of *C. ficifolia* to FOC.

### Phytohormone and TFs involved in defense of C. ficifolia to FOC

Phytohormones play important role in defense and immune responses. Although SA, JA, and ET are the main phytohormones associated with immune responses against pathogens, ABA, gibberellic acid, Cytokinin, and auxin, are also key parts of the defense response of plants [10]. A number of DEGs related to hormone signaling were found in *C. ficifolia* after FOC infection (Tables 1 and 2). ET is the principal modulator of the defense response to pathogens. The synthesis of ET begins with the amino acid methionine, which is first converted into S-adenosylmethionine and then into 1-aminocyclopropane-1-carboxylic acid (ACC). ACC is then converted into ET by ACC oxidase (ACO) [24]. Genes encoding ACO can be transcriptionally up-regulated, resulting in ET biosynthesis activation in plants challenged by pathogens. Genes encoding ACO are up-regulated in Arabidopsis infected with *Botrytis cinerea* [10]. In tomato, ET is also required for the xylem occlusion response to counter the further spread of *F. oxysporum* f.sp*. lycopersici* [11]. A DEGs annotated as ACO was both up-regulated by FOC infection in 2d-vs-ck and 4d-vs-ck (Table 1). Another ACO was up-regulated only in 4d-vs-ck. Auxin and JA synergize to promote resistance to necrotrophic pathogens [22]. Seven DEGs with annotations related to auxin were both up-regulated by FOC infection in 2d-vs-ck and 4d-vs-ck (Table 1). ABA is closely linked to resistance to a variety of abiotic stress, especially drought and salinity. The exogenous application of ABA can reduce the resistance of potato to *Phytophthora infestans* and the resistance of tobacco to *Peronospora tabacina* [25, 26]. These finding indicated that ABA can depress plant resistance to pathogens, especially fungi. ABA signaling in plants involves perception by a receptor complex that is formed by PYRABACTIN RESISTANCE 1 (PYR) and PYR1-LIKE (PYL) proteins [27]. The ABA receptor PYL4 was both up-regulated by FOC infection in 2d-vs-ck and 4d-vs-ck (Table 1). The ABA receptor PYL8 was up-regulated by FOC infection only in 4d-vs-ck. These results indicated that the ET, auxin, and ABA signal transduction pathways may play a pivotal role in the response to FOC infection.

Biotic stresses trigger a wide range of plant responses, TFs function in the promoter region of stress-related genes; the induction or reduction the expression of these genes may change plant tolerance to biotic stress [28]. Nearly 30 DEGs were TFs and were up-regulated by FOC infection in 2d-vs-ck and 4d-vs-ck. Most of them belonged to the MYB, bHLH, WRKY, NAC, LUX and GLK TF families and presented high increment or decrement after FOC infection (Tables 1 and 2). MYB TFs play an essential role in defense responses in plants. *Sp*MYB (*Solanum pimpinellifolium* L3708) expression is significantly induced after infection by *F. oxysporum*. Overexpression *Sp*MYB in tobacco increased resistance to *F. oxysporum* and the transgenic plants had lower malonaldehyde content but increased peroxidase, superoxide dismutase, and phenylalanine ammonia-lyase activities [29]. WRKY TFs are the global regulators of plant defense signaling. A previous study on two chickpea (*Cicer arietinum* L.) genotypes with contrasing resistance against *F. oxysporum* f. sp. *ciceri* Race1 (Foc1) demonstrated that *Ca*WRKY40 triggered defense to Foc1. In chickpea, overexpressed CaWRKY40 induced resistance to Foc1 by binding to promoters and positively regulated the transcription of *Ca*Defensin and *Ca*WRKY33 [30].

Many reports have indicated that NAC TFs are the principal modulators of plant defense, and systemic acquired resistance [31]. Numerous examples have shown that the expression of the NAC gene change after pathogen infection. The *StNAC* (*Solanum tuberosum*) gene was induced after *Phytophthora infestans* infection [32]. In rice seedlings, 19 and 13 NAC genes were up-regulated after RSV and RTSV infection, respectively [33]. The NAC domain-containing protein 79 was up-regulated by FOC infection only in 4d-vs-ck (Table 2). A number of NAC proteins activate PR genes to regulate plant defense responses [31]. In Arabidopsis, the overexpression of ATAF2, the NAC TF, increased susceptibility to *F. oxysporum* by inhibiting PR genes expression [34]. In Arabidopsis, cold stimulated the activation of the NAC TF NTL6 which induced PR genes and enhanced disease resistance [35]. Three PR genes were found in *C. ficifolia* only in 4d-vs-ck. One was down-regulated and two were up-regulated. However, only two up-regulated PR genes were up-regulated at the transcription and translation levels in 4d-vs-ck (Additional file 6). These results indicated that NAC TFs may regulate defense responses to FOC by activating PR proteins in *C. ficifolia*.

### Wax biosynthetic process and photosynthesis are repressed by FOC

After FOC infection, the numbers of genes related to wax biosynthesis and photosynthesis decreased. The wax biosynthesis was significantly reduced by FOC in 4d-vs-ck. (Table 2). Two genes that were annotated as omega-hydroxypalmitate O-feruloyl transferase were down-regulated (Table 2). Omega-hydroxypalmitate O-feruloyl transferase is a pivotal enzyme in the biosynthesis of unsaturated fatty acids, which are the precursors of wax [36]. Seven genes that were annotated as 3-ketoacyl-CoA synthase (KCS) were also down-regulated (Table 2). In potato, stably silencing the KCS gene *StKCS6*, through RNA interference, decreased the accumulation of peridermal wax [37]. Although FOC infects the plant from root, FOC infection down-regulated 22 DEGs that were related to photosynthesis in 4d-vs-ck. Similar situation was encountered in chickpea infected by *F. oxysporum* f. sp. *ciceri* race 1 (FOC1). The photosynthetic stability of susceptible plants was hampered by the down regulation key photosynthetic genes and the photosynthetic stability of resistant chickpea also decreased at later time points [38].

Three photosynthesis-related DAPs show increased accumulation at 2dpi and 4dpi. This is contrary to the transcriptome results, and one explanation is that photosynthesis can help plants defect pathogens through providing of carbon skeleton and energy [39]. The same pattern was observed in the proteomic profile of *Pinus monticola* infected by *Cronartium ribicola* in compatible and incompatible interaction [40]. In contrast, photosynthesis-related proteins showed increased accumulation in the early stages (72 h) and decreased accumulation in the later stages(45 d) of cocao infected by the pathogen *Moniliophthora perniciosa* [41].

### Oxidative-redox proteins are induced to resist FOC

The rapid accumulation of reactive oxygen species (ROS) is the earliest typical event in a plant–pathogen interaction [42, 43]. ROS are toxic for both host and pathogens, therefore, the balance between production and removal of ROS are important during stress response [44]. Plants use anti-oxidative enzymes to eliminate ROS. Six oxidative-redox proteins including ferredoxin, catalase isozyme and peroxidase were both up-regulated after FOC infection at 2dpi and 4dpi. In many plant species, up-regulated peroxidases are in line with resistance [45]. *Moniliophthora perniciosa* is the causal agent of cacao (*Theobroma cacao* L.) witches’ broom disease (WBD). The cacao genotypes with WBD resistance showed up regulation of oxidative stress proteins twice as large as sensitive genotypes, particularly in proteins related to ROS detoxification [41]. It was also demonstrated that the upregulation of detoxification proteins promoted resistance of *Citrus* genotypes to Huanglong disease [46]. These results reveal that accumulation of oxidative-redox proteins plays an important role in the resistance of *C. ficifolia* to FOC.

## Conclusions

**Fig. 5:**
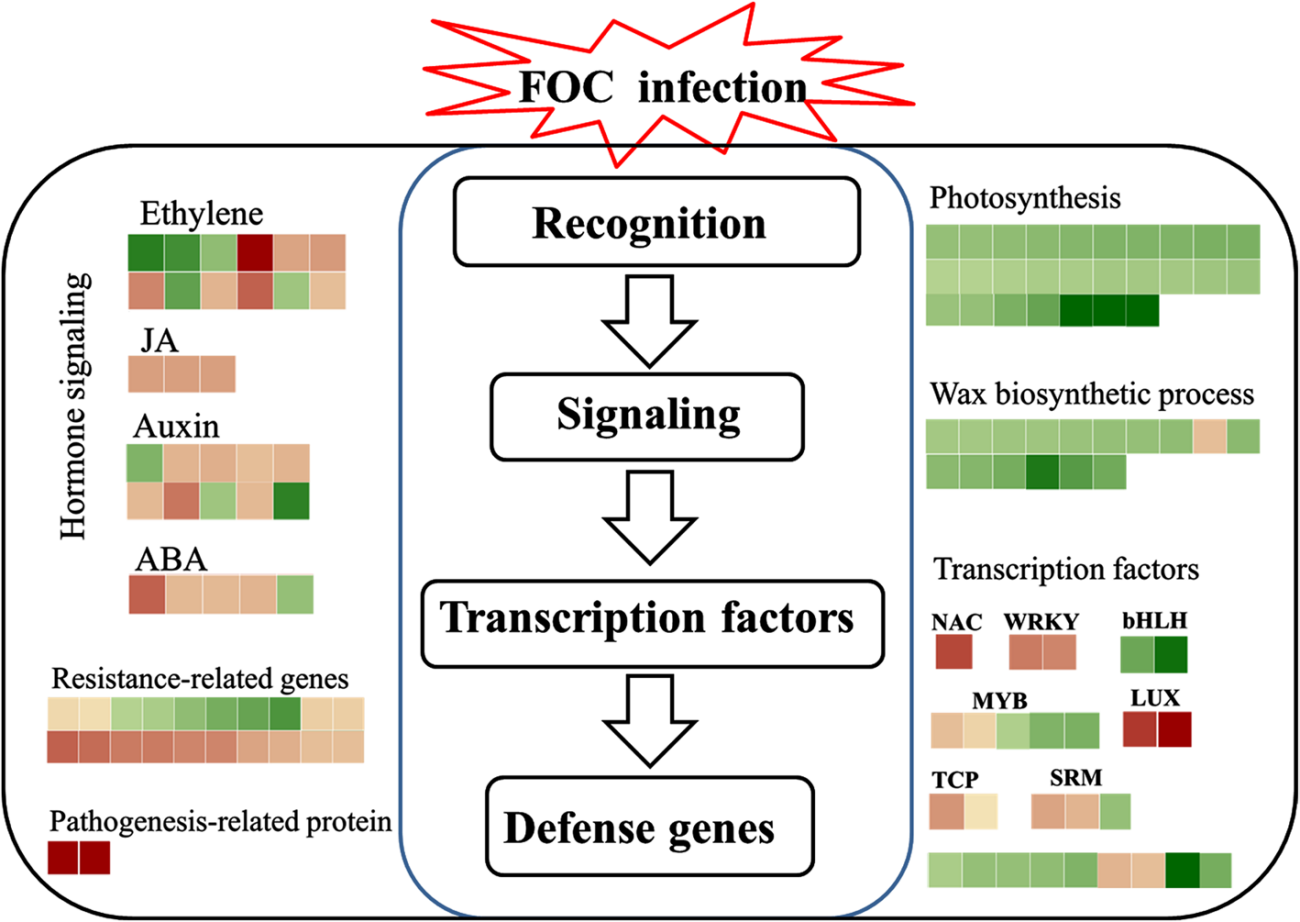
Biotic stress–related genes altered in response to FOC infection. Genes that were differentially expressed between control and infected leaf are indicated by colored squares, based on their pattern of expression at the gene level at 2d and 4d. Negative values represent repressed genes (green) and positive values represent up-regulated genes (red)

This study is the first to determine the response of *C. ficifolia* leaves to FOC infection at the transcription and translation levels. It revealed that FOC infection activated phytohormone signaling and TFs but inhibited wax biosynthesis and photosynthesis (Fig. 5). The accumulation of oxidative-redox proteins also plays an important role in the resistance of *C. ficifolia* to FOC. The results provide new information regarding the processes of *C. ficifolia* leaf resistance to FOC and will contribute the rootstock breeding of cucumber with resistance to fungal pathogen.

## Methods

### Cultivation of plants and pathogenic fungi

This experiment was conducted in a greenhouse at Yunnan Agricultural University, Kunming, and southern China. *C. ficifolia* Bouché was used as the material in this study. The seeds were collected from local growers in Kunming, Yunnan Province. The seeds identified by Prof. Zhengan Yang, and then kept in the laboratory for long-term use. All *C.ficifolia* seeds were initially sterilized in 10% hydrogen peroxide for 1 h, rinsed five times with sterile deionized water, and then subjected to accelerated germination in a constant temperature incubator at 28 °C in the dark. After germination, the seeds were transplanted into pots containing peat soil and perlite (V:V = 1:1) and kept in a plant growth chamber under a 12 -h -light/12 –h -dark photoperiod and at the ambient temperature of 25 °C ± 2 °C.

FOC was provided by the Department of Plant Nutrition, Nanjing Agricultural University. The strains were well maintained and were transferred into potato dextrose agar medium for 7 days before inoculation. Agar disks cut from the 7-day-old cultures were filtered through two layers of sterile gauze to remove mycelial fragments and then diluted to the concentration of 1 × 10^8^ conidia/mL with sterile distilled water.

When the third true leaf had emerged, *C.ficifolia* seedlings were inoculated with 10 mL of endoconidia suspension (fungal infection) or with 10 mL of sterile water (control) through root dipping. Endoconidia suspension (10 mL) was added into the pots of the seedlings with fungal infection to ensure successful FOC infection. Leaves were harvested from the control and infected plants at 2 and 4 dpi for RNA extraction. The leaves from three seedlings were harvested together as one sample, and three biological replicates were used for RNA-seq, iTRAQ and RT-PCR analyses. They were immediately frozen in liquid nitrogen and stored at − 80 °C.

### RNA sequencing and DEGs identification

Total RNA was isolated by using TRIzol reagent (Invitrogen, USA) in accordance with the manufacturer’s instructions. A minimum of 50 mg of the total RNA of each sample were transported to oeBiotech (Shanghai, China) for sequencing.

Equal amounts of the total RNA of the control and infected *C. ficifolia* plants at 2 and 4 dpi were pooled in a combined sample for PacBio library construction and sequencing. Three biological replicates were used for PacBio sequencing. Twelve cycles of PCR amplification were performed by using PrimeSTAR GXL DNA Polymerase (Clontech, USA). After purification with AMPure PB Beads, the cDNA products were then used for the construction of SMRTbell template libraries. One SMRT cell was sequenced on a PacBio Sequel instrument by using a Sequencing kit 2.1 with 10 h movie recordings. Sequencing reads were subjected to circular consensus sequences by using SMRT Analysis Software (https://www.pacb.com/products-and-ser vices/analytical-software/devnet/). PacBio reads were classified into full-length and nonfull-length sequences, and then were corrected with Illumina data generated from the same *C. ficifolia* RNA samples by using LoRDEC [47]. The isoform was clustered to obtain unigenes (identity = 98%) by using CD-HIT [48].

Nine cDNA libraries (the control and infected plants at 2 and 4 dpi with three biological replicates were named ck1, ck2, ck3, 2d-1, 2d-2, 2d-3, 4d-1, 4d-2, and 4d-3)were constructed for RNA-seq on the Illumina HiSeq™ 2500 platform. Reads appearing in three biological replicates of Illumina were mapped to PacBio sequence for further analysis and normalized to obtain the normalized gene expression level on the basis of Fragments Per kb per Million reads(FPKM) by using bowtie2 [49], and the formula is shown as follows:

FRKM(A) = 10^9^C/NL.

Where FPKM(A) is the expression of gene A, C is the number of reads that are uniquely aligned to gene A, N is the total number of reads that are uniquely aligned to all genes, and L is the number of bases in gene A.

DEGs between the infection and control were screened on the basis of the general method with expression changes no less than two folds (log2 ratio ≥ 1) and false discovery rates (FDRs) < 0.05 [50]. Gene set enrichment analysis with GO data was performed on these DEGs by using Goseq [51]. These DEGs were mapped to KEGG pathway to identify key genes involved in resistance to FOC [52]. The raw Illumina sequencing data had deposit in SAR with the number SRX9738784 to SRX9738792, the raw PacBio sequencing data had deposit in SAR with the number SRX9778938 and can be obtained directly through the link:https://www.ncbi.nlm.nih.gov/sra/?term=cucurbita+ficifoliaand. The commands and parameters used for running bioinformatics programs/pipelines in this manuscript are shown in Additional file 7.

### Protein extraction and DAPs identification

The TCA/acetone method was used to extract the total protein of each sample. The samples were ground into powder in liquid nitrogen. The powder was added to phenol extraction buffer, incubated for 10 min, shacked for 40 min, and centrifuged at 15,000 × *g* for 1 min. The precipitate was dried and resuspended in phenol extraction buffer. Then, the protein was extracted in accordance with the phenol extraction method [21].

iTRAQ analysis was completed at oeBiotech (Shanghai, China). An iTRAQ 8-plex kit was used to label digested protein samples according to the manufacturer's protocol. All of the samples were pooled and separated via liquid chromatography after labeling. Mass spectrometer data were acquired with a Triple TOF 5600 System (AB SCIEX, USA).

Protein species were identified by using Mascot software (version 2.3.02, Matrix Science Inc., 231 Boston, MA, USA) against PacBio sequences. Peptides with significant scores (≥ 20) at the 99% confidence interval were considered as identified. The identified peptide sequences were then assembled into a set of accurately identified proteins. The quantitative protein species ratios were weighted and normalized by using the default parameters of the Mascot software package. A twofold cutoff value was used to identify DAPs with *P* value < 0.05 [53].

The proteomics data have been deposited to the ProteomeXchange Consortium via the PRIDE partner repository under the dataset identifer PXD028075.

### Quantitative RT-PCR analysis

Total RNA was isolated from the infected and control of *C. ficifolia* as mentioned above. Reverse transcription process and qRT-PCR was carried out following previously described methods [54]. The specific primers used for real-time PCR are shown in Additional file 8.

## Supplementary Information

**Additional file 1.** DEGs in C. ficifolia leaf after infection by FOC at 2 dpi compared with control.

**Additional file 2.** DEGs in C. ficifolia leaf after infection by FOC at 4 dpi compared with control.

**Additional file 3.** DAPs in C. ficifolia leaf after infection by FOC at 2 dpi compared with control.

**Additional file 4.** DAPs in C. ficifolia leaf after infection by FOC at 4 dpi compared with control.

**Additional file 5.** DEGs and DAPs in C. ficifolia leaf after infection by FOC at 2 dpi.

**Additional file 6.** DEGs and DAPs in C. ficifolia leaf after infection by FOC at 4dpi.

**Additional file 7.** Commands and parameters used for running bioinformatics programs/pipelines.

**Additional file 8.** Primer sequences used for qRT-PCR.

## Data Availability

The raw PacBio and Illumina sequencing data had deposit in SRA with the number SRX9778938 and SRX9738784 to SRX9738792. The proteomics data have been deposited to the ProteomeXchange Consortium via the PRIDE partner repository under the dataset identifer PXD028075.

## Abbreviations

ABA: Abscisic acid
ACC: 1-Aminocyclopropane-1-carboxylic acid
ACO: 1-Aminocyclopropane-1-carboxylic acid oxidase
DAPs: Differentially accumulated proteins
DEGs: Differentially expressed genes
dpi: Day post infection
ET: Ethylene
FDR: False discovery rate
FOC: Fusarium oxysporum f. sp. Cucumerinum
iTRAQ: Isobaric tag for relative and absolute quantitation technology
JA: Jasmonic acid
ROS: Reactive oxygen species
SA: Salicylic acid
TFs: Transcription factors
