# Supplementary material for: Transcriptomic and proteomic analyses of Cucurbita ficifolia Bouché (Cucurbitaceae) response to Fusarium oxysporum f.sp. cucumerium
Source: BMC Genomics. 2022 Jun 13;23(Suppl 1):436. doi: 10.1186/s12864-022-08674-7 (PMC9190096; doi:10.1186/s12864-022-08674-7)
Supplement: Supplementary file 7 — Additional file 7. Commands and parameters used for running bioinformatics programs/pipelines. [file 12864_2022_8674_MOESM7_ESM.docx]

Commands and parameters used for running bioinformatics programs/pipelines in this manuscript

CD-HIT (cluster analysis for obtaining unigenes ):

>cd-hit-est -i input.fasta -o output-cdhit -T 10 -M 200000

bowtie2（aligning sequencing reads to long reference sequences）:

>bowtie2-build reference sequences.fa reference sequences.fa

>bowtie2 -p 6 -3 5 --local -x ref sequences -1 example_1.fastq -2 example_2.fastq -S example.sam

>samtools sort example.sam > example.bam

DESeq (differential expression analysis), padj<0.05 and |log2FoldChange|>1:

>diff_gene_deseq2 <-subset(res,padj < 0.05 & (log2FoldChange > 1 | log2FoldChange < -1))

GoSeq (enrichment analysis ), the FDR (adjust P-value) < 0.05

>getgo(genes, ref sequences, id,fetch.cats=c("GO:CC","GO:BP","GO:MF"))

getlength(genes, ref sequences, id)

>nullp(DEgenes, ref sequences, id, bias.data=NULL,plot.fit=TRUE)

>goseq(pwf, genome, id, gene2cat = NULL,test.cats=c("GO:CC", "GO:BP", "GO:MF"),method = "Wallenius", repcnt = 2000, use_genes_without_cat=FALSE)

>enriched.GO<-pvals [pvals$over_represented_pvalue<0.05,]
